# In-Line Defect Detection on Painted Surfaces in Motion Using Template Matching

**DOI:** 10.3390/s26217038

**Published:** 2026-11-07

**Authors:** Rodrigo Rito, Ana M. Rocha, João Fontoura, Luis Rino, Paulo Antunes

**Affiliations:** Institute for Nanostructures, Nanomodelling & Nanofabrication (i3N) and Physics Department, University of Aveiro, Campus Universitário de Santiago, 3810-193 Aveiro, Portugal; rodrigorito@ua.pt (R.R.); amrocha@ua.pt (A.M.R.); jplfontoura@ua.pt (J.F.); lrino@ua.pt (L.R.)

**Keywords:** quality control, machine vision, dark field illumination, template matching, logistic regression model, defect detection

## Abstract

Quality control of exterior surfaces, particularly painted finishes, is a determining factor in the perceived quality of a device, which impacts the purchasing decision. Automating this process reduces potential human mistakes and improves efficiency. In this work, we propose an automatic defect-detection system for painted surfaces of water heater covers, consisting of an image acquisition setup and a defect-detection model. The proposed approach focuses on crater defects, one of the most frequent and challenging defect types to identify. A dark-field illumination-based image acquisition system was developed to enhance defect visibility while accounting for production-line constraints, including line motion velocity, ensuring its applicability in real industrial environments. The proposed setup enabled the acquisition of high-quality images with clearly defined surface defects. The proposed approach was focused on the binary classification of water heater covers as defective or non-defective based on the presence of crater defects. Defect identification is based on a template matching algorithm, and a logistic regression model is applied for the classification. The developed system demonstrated promising performance, achieving an accuracy of 95.65%, a of 90.00%, and a recall of 100%. All defective samples were correctly identified, with only one non-defective surface misclassified.

## 1. Introduction

The perceived quality of a device significantly impacts the purchasing decision. Painting is therefore widely used to enhance exterior appearance, as well as to ensure surface protection, but painted surfaces are particularly susceptible to defects. One of the most common painting techniques used in the metal coating industry is powder coating. In this technique, charged particles are electrostatically sprayed onto the metal surface, granting a smooth and long-lasting finish to the product [1,2]. This process presents numerous advantages, such as coating durability, energy efficiency, and compliance with environmental regulations [2,3]. However, in the final product, several visual defects can appear due to different causes, including surface contamination or moisture, which can lead to crater and pinhole formation, and excessive or incorrect powder application, which can lead to “orange peel” or lack of paint [1,2].

For this reason, the quality control of the exterior appearance of devices plays a critical role, yet automating this process remains a challenge for the industry. In many industries, the inspection of painted surfaces is carried out through subjective human evaluation based on qualitative judgments, which requires specialized workers and makes it difficult to ensure consistent minimum quality standards [4,5]. Automation of this process can reduce the resources required for defect correction by minimizing possible human errors. In this context, machine vision systems have been widely adopted in quality inspection processes. These image-based techniques typically involve the acquisition of images and their subsequent processing for defect detection. The inspection system used should consider the specifications of the product to be inspected, such as the reflective nature and curvature of the surface in the analysis, the types of defect to analyze (such as shape, depth, size), the lighting conditions and the space required to implement the inspection apparatus [6]. Furthermore, the inspection process can be static or dynamic, depending on the acquisition conditions, such as the inspection of moving objects in a production line under factory conditions [7].

In this work, we propose an automatic defect-detection system for the painted surfaces of water heater covers. These surfaces are coated via powder coating techniques, exhibit specular reflective properties, and may present some defects, as discussed above. Here, we will focus on detecting the presence of crater-type defect (also referred as dimples) [8]. These defects are among the most frequent and challenging to identify due to their small size, ranging from 1 mm to 3 mm in diameter, and low contrast under conventional illumination. The proposed solution includes an image acquisition system and a defect-detection model that considers production-line constraints, making it suitable for real production environments.

Several illumination approaches can be used for image acquisition of specular surfaces. Among them, deflectometry-based techniques are the most commonly used in a wide range of applications [5,6,9,10,11]. This technique is based on the observation of known structured illumination patterns, such as sinusoidal fringes, displayed on a screen (e.g., a LCD screen) and reflected by the surface under inspection toward a camera. Any distortions in the acquired reflected pattern correspond directly to variations in surface slopes or defects [6,9,11]. Therefore, in deflectometry, the illumination and observation directions must comply with the law of reflection, which imposes significantly stricter requirements on the geometric configuration of the setup. This requirement becomes particularly challenging in industrial environments, such as production lines, where space is limited and objects are in motion. Furthermore, although deflectometry has high sensitivity to surface slope variations, particularly near the transition from dark to bright fringes, its spatial resolution may not be sufficient to detect small or localized defects [8].

Another illumination approach used for image acquisition of specular surfaces is dark-field illumination, which enhances surface topography by suppressing specular reflections emphasizing scattered light from surface irregularities [8,12,13]. In the dark-field technique, the surface is illuminated at a low incident angle, and the camera is positioned outside the specular reflection direction, typically perpendicular to the surface. For a defect-free flat surface, the resulting captured image is a uniform low gray image since little to no light is scattered toward the camera. However, in the presence of a defect, the incident light is scattered in multiple directions and can reach the camera, producing a brighter region in the image. This technique has already been applied in different fields, including the inspection of the surfaces of car bodies [4] and optical elements [14].

Recently, the combination of images acquired under different illumination strategies has gained attention in vision-based inspection systems [11,12,15]. By exploiting complementary information from multiple illumination techniques, these systems achieve a more comprehensive and reliable surface defect characterization than single-modality approaches. However, they also introduce additional complexity and computational cost, as well as the potential for redundancy.

This work was focused on detecting the presence of crater-type defects; therefore, dark-field illumination was used. This illumination technique is particularly effective in highlighting surface discontinuities with smooth and reflective sidewalls, such as craters [8], enabling reliable detection without the need for additional illumination techniques.

In automated visual inspection, image processing and analysis provide the means to detect, localize, and characterize surface defects from acquired images. A robust image-processing system is thus crucial to achieving accurate and repeatable performance [16]. Deep learning-based algorithms have demonstrated effective performance in defect detection and classification, including in specular painted surfaces [10,11,17]. However, such methods are computationally expensive and require large labeled training datasets, which are often impractical for painted surfaces inspection due to the low occurrence rate of defects. Thus, for a simple task such as the binary classification of specific defects, traditional methods are an efficient alternative, providing the required decision-making process with lower computational cost.

Here, we propose the use of a logistic regression-based algorithm combined with a template matching method to detect crater defects and perform binary classification of water heater covers as either defective or non-defective. Template matching is a widely used method in object recognition, enabling the extraction of specific features and the quantitative assessment of similarity between images [18]. This type of method is commonly applied in tasks where a predefined pattern must be located within a larger image, making it particularly effective for feature localization and defect detection, such as smartphone camera defect detection [19], synthetic aperture radar image registration [20], structural health monitoring [16], circuit board inspection [21], and printing defect detection [22].

## 2. Image Acquisition System

**Figure 1:**
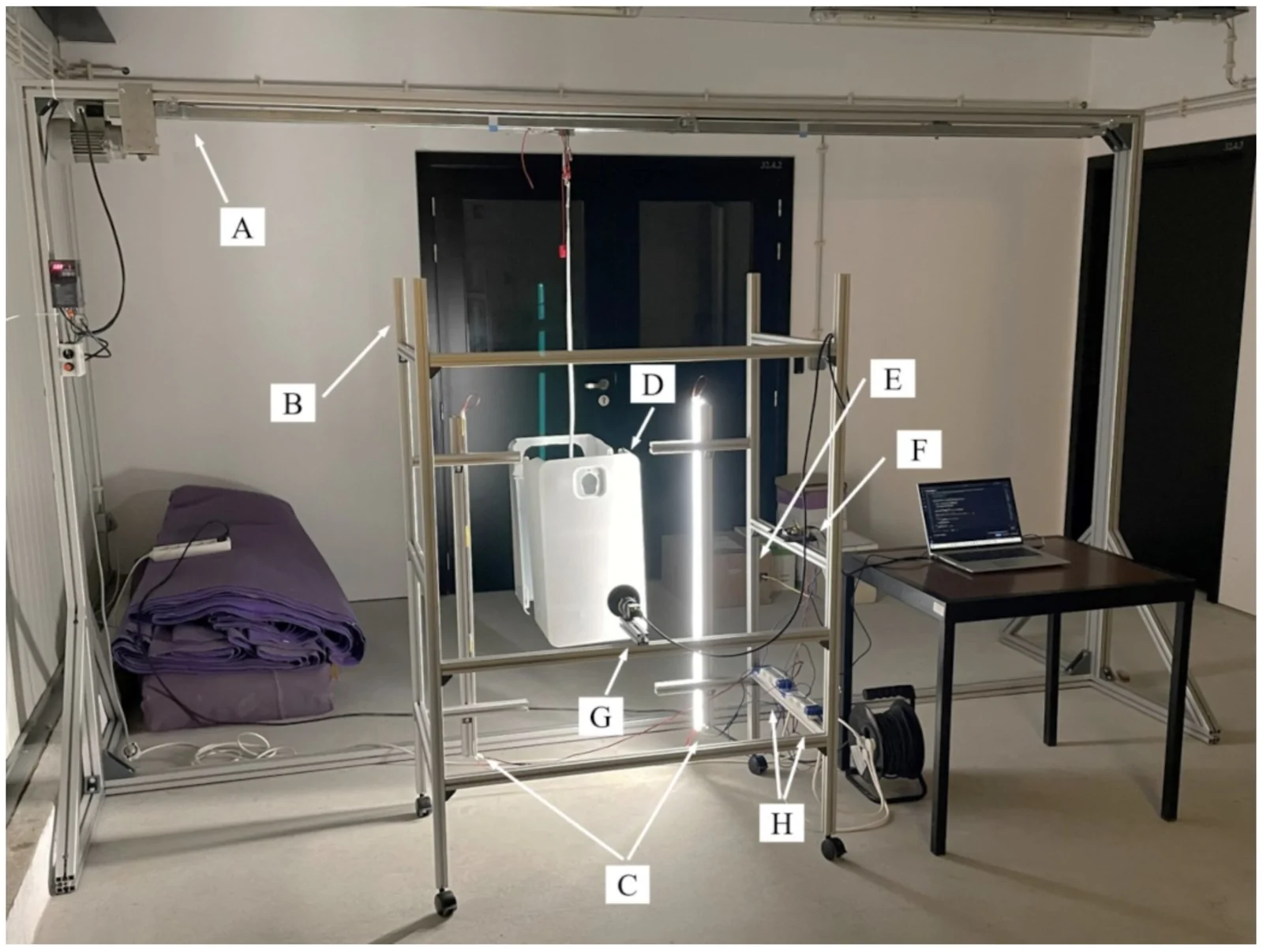
Production line simulator (A) and image acquisition system developed: support structure (B), LED illumination (C), water heater cover (D), motion sensor (E), control system (F), machine vision camera (G), and LED’s power supply (H).

The machine vision-based inspection system proposed in this study is designed to be used in a production painting line for water heater covers with maximum dimensions of approximately 700×400 mm. Therefore, production-line constraints, including space limitations, lighting conditions, and line velocity, were considered in the design of the image acquisition system. To match the real operating conditions, a production-line simulator (indicated as A in Figure 1) was developed to control the movement of the water heater covers. The simulator operated at a velocity of 3.2 m/min, corresponding to the maximum velocity of the production line.

The proposed image acquisition system applies dark-field lighting to illuminate the painted surfaces of water heaters, as discussed in Section 1. Using this technique, the camera is positioned perpendicular to the surface being analyzed, while a grazing light source is placed at a low angle of incidence. With this approach, defect visibility depends on the orientation of surface irregularities relative to the illumination direction. However, crater-type defects are approximately symmetrical and can be detected under either left-side or right-side illumination. Nevertheless, both illumination directions are necessary to ensure complete and uniform visualization of the heater covers surface. Thus, two images are captured per surface, one for each illumination side, ensuring visualization of the entire surface.

**Figure 2:**
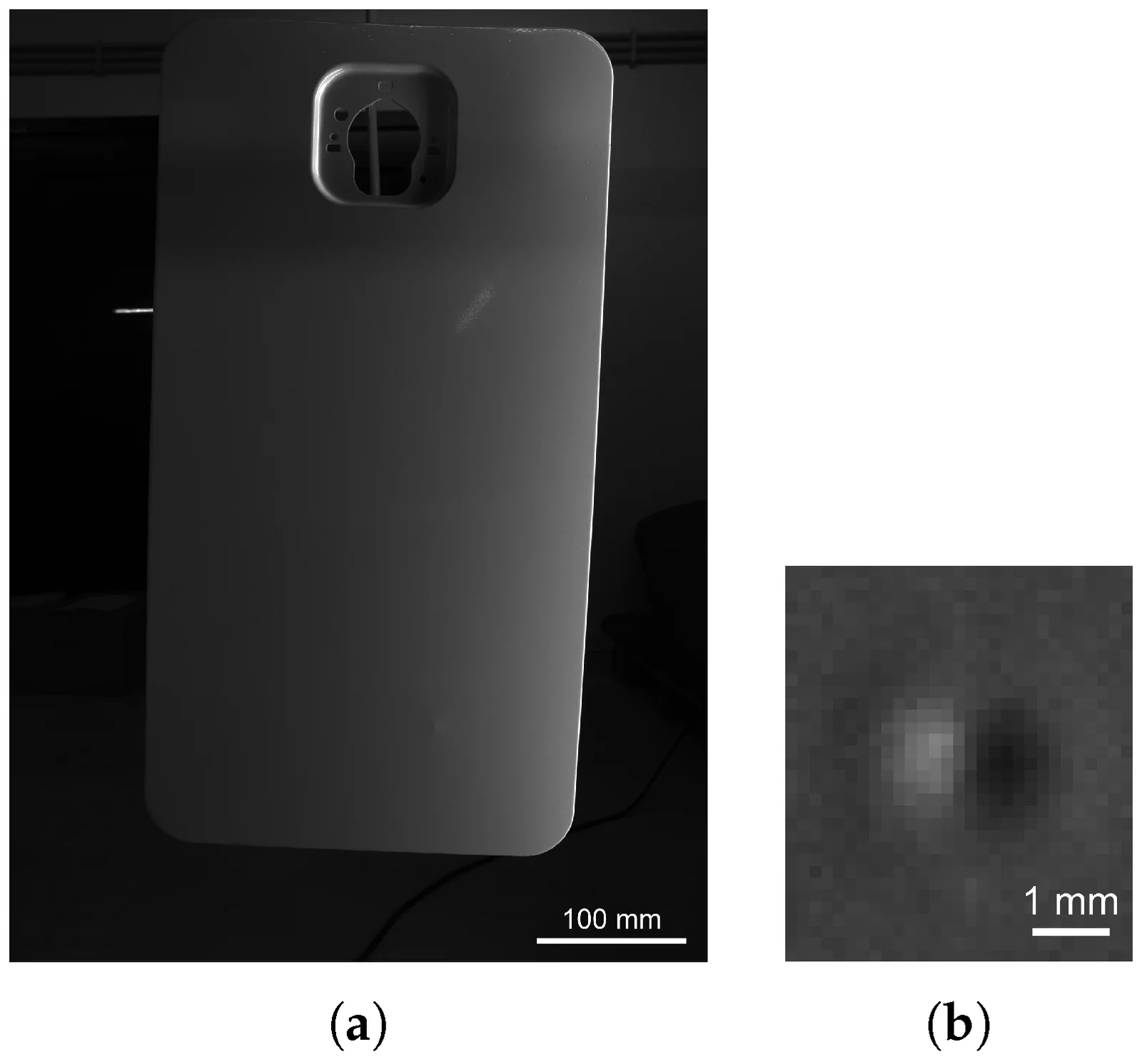
Example of an acquired image of a water heater cover measuring 580×310 mm (**a**). A close-up of a crater-type defect, with around 2 mm of diameter (**b**).

The developed setup, shown in Figure 1, consists of three 1 m long LED stripes positioned in each side of the cover, each providing a luminous flux of 7800 lm, and a machine vision camera (IDS, model U3-31L0CP-M-GL, Obersulm, Germany) with a resolution of 12.4 MP (4096×3000 pixels), equipped with a lens with a focal length of 8 mm (IDS, model 20M11-C0828 8mm, Obersulm, Germany). The lens was selected to capture the entire surface in a single image within the working distance available on the production line, as presented in Figure 2a. The camera resolution was selected to ensure sufficient spatial detail for visualizations of the smallest defects. To synchronize image acquisition with the movement of the water heater covers in the production-line simulator and with the illumination position, an infrared motion module sensor (Seeed, ITR9909, Frankfurt, Germany) was used to detect the presence of the surface. An Arduino microcontroller processes the sensor signal and triggers both the illumination and the image acquisition. To minimize motion blur and ensure sharp images with clear visibility of surface defects, a compromise between camera aperture and exposure time was established. The camera exposure time was set to an optimized value of 15 ms. Figure 2 presents an image acquired with the proposed system (a), and a close-up of a crater-type surface defect that shows its visibility (b). Reflections from the ambient illumination are also visible in this image; however, they do not affect the visualization of the defects.

## 3. Defect Detection Methodology

As outlined in Section 1, this work focuses on detecting crater-type defects, which are among the most common and critical defect types. The defect-detection methodology developed comprises defect detection followed by a binary classification and decision process. The identification of defects is achieved through a template matching algorithm based on normalized cross-correlation, while a logistic regression (LR) classifier is used for classification. The decision is made based on the combined results obtained from the pair of images acquired for each heater cover.

### 3.1. Template Matching Technique

Template matching involves calculating a similarity (or distortion) measure at every location in an image to assess its similarity to a predefined template. This technique uses a sliding-window approach, where a target pattern, represented by a small reference image (template), is systematically scanned across a larger input image. By evaluating regions of equivalent dimensions, the process identifies the region of interest exhibiting the highest degree of similarity, enabling the target pattern to be detected [18,23].

#### 3.1.1. Cross-Correlation

The most straightforward approach to measuring the degree of similarity is to consider the sum of the squared differences between the template, *t*, at the coordinates (*u*, *v*), and the corresponding region in the image under analysis, *f*, as shown in Equation (1) [24].

(1) $$d_{f,t}^{2}\left(u,v\right)=\sum_{x,y}{\left[f\left(x,y\right)-t\left(x-u,y-v\right)\right]}^{2}$$

**Figure 3:**
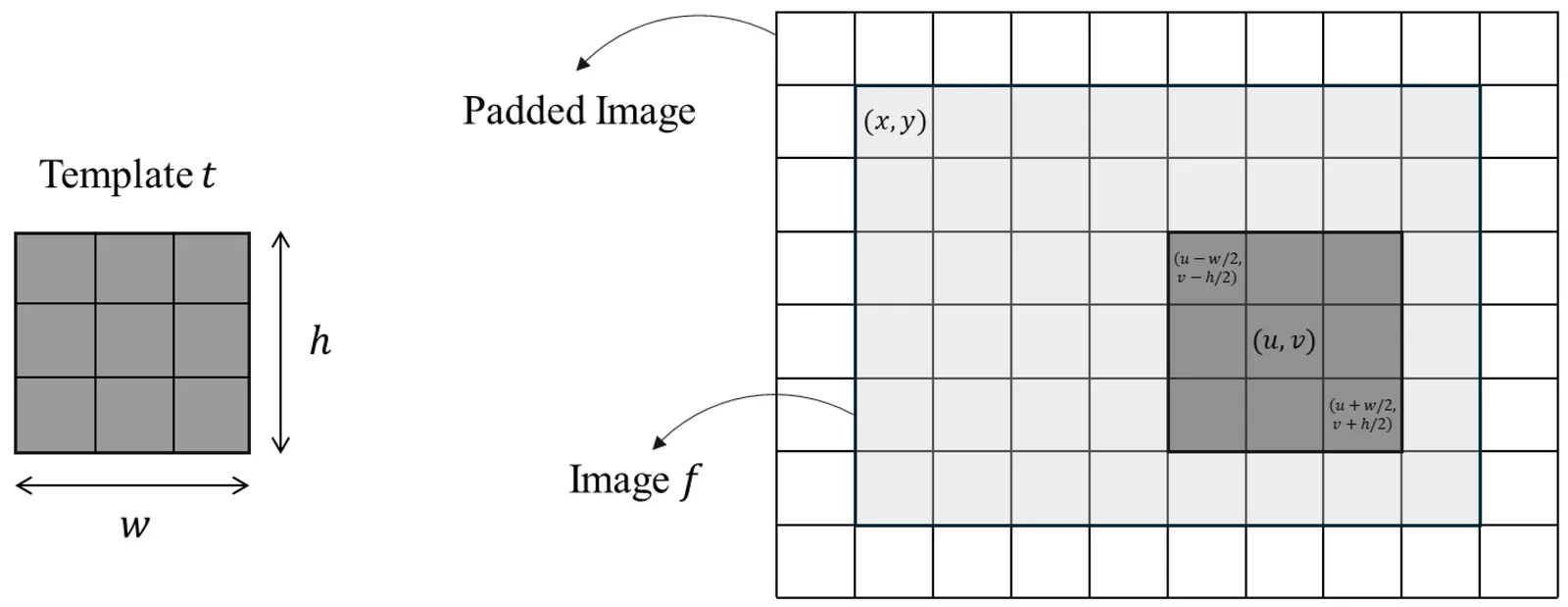
On the left, the template, *t*, and on the right, the cross-correlation method in image *f*. The template is located on a random location (u,v) of the image.

Therefore, *x* ranges from [u−w/2,u+w/2] and *y* ranges from [v−h/2,v+h/2], where *w* and *h* denote the template’s width and height, respectively, as shown in Figure 3. To ensure that the center pixel of the template aligns with every pixel of the image in the analysis, the image needs to be padded with zeros [18,23].

By expanding the difference, $d_{f,t}^{2}$, we obtain:

(2) $$d_{f,t}^{2}\left(u,v\right)=\sum_{x,y}\left[f^{2}\left(x,y\right)-2f\left(x,y\right)t\left(x-u,y-v\right)+t^{2}\left(x-u,y-v\right)\right]$$

Since for a given template, *t*, the term $t^{2}\left(x-u,y-v\right)$ is constant, and assuming that the term $f^{2}\left(x,y\right)$ is approximately constant, the remaining term can be considered as the correlation term, which is defined as:

(3) $$c\left(u,v\right)=\sum_{x,y}\left[f\left(x,y\right)t\left(x-u,y-v\right)\right]$$

This quantifies the similarity between the template, *t* and the image, *f*, at the coordinates (*u*, *v*) [18,24].

The complete cross-correlation map is obtained by sliding the template over the entire image, computing the correlation at each pixel. The highest cross-correlation coefficient value corresponds to the template’s best match [18].

However, if the image presents strong intensity variations, the use of the cross-correlation term presented in Equation (3) can fail. For example, if the image contains a bright spot, the correlation between the template and this area may be higher than the correlation between the template and the region of the image that matches it exactly [24]. To address this limitation, an improved cross-correlation approach is used, known as normalized cross-correlation.

#### 3.1.2. Normalized Cross-Correlation

By normalizing the correlation term, the resulting metric becomes invariant to global brightness changes, thereby mitigating the impact of image intensity variations [18], as demonstrated in Equation (4):

(4) $$\gamma \left(x,y\right)=\frac{\sum_{x,y}\left[f\left(x,y\right)-{\bar{f}}_{u,v}\right]\left[t\left(x-u,y-v\right)-\bar{t}\right]}{\sqrt{\sum_{x,y}{\left[f\left(x,y\right)-{\bar{f}}_{u,v}\right]}^{2}\sum_{x,y}{\left[t\left(x-u,y-v\right)-\bar{t}\right]}^{2}}},$$

where $\bar{t}$ is the mean of the template and ${\bar{f}}_{u,v}$ is the mean of f(x,y) in the region under analysis [24]. The normalized correlation spans the interval [−1,+1], with values close to +1 indicating significant similarity between images, and values close to −1 indicating that the images are completely different.

### 3.2. Normalized Cross-Correlation Algorithm Implementation

**Figure 4:**
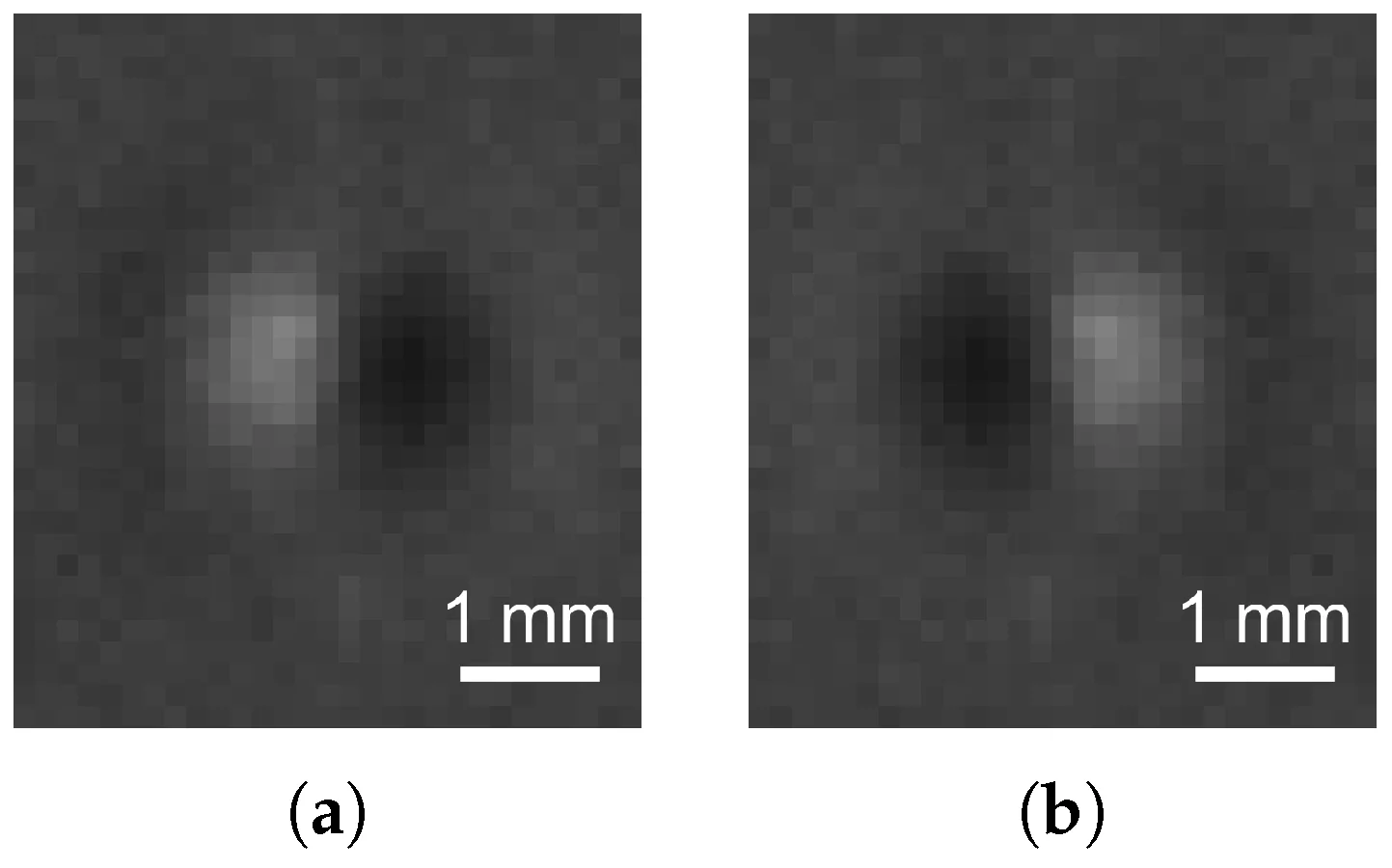
Template selected for the defect type crater: original (**a**) and flipped version (**b**).

The first step to implement the template matching using normalized cross-correlation (NCC) is to select a template representative of the defect type being analyzed. Several representative crater templates were evaluated and produced similar results; therefore, the template shown in Figure 4a was selected as a representative example. As observed, the template has a very characteristic shape, resulting from the illumination setup, in which the light source is positioned to the right of the surface in this case. In the opposite case, where the light source is positioned to the left of the surface, the shape of the defect template remains unchanged but the illumination is reversed. Thus, the same template can be used for both cases, but flipped horizontally to improve match accuracy in the cross-correlation analysis. In this way, two templates are used, one of which is the horizontal flip of the other, as shown in Figure 4.

**Figure 5:**
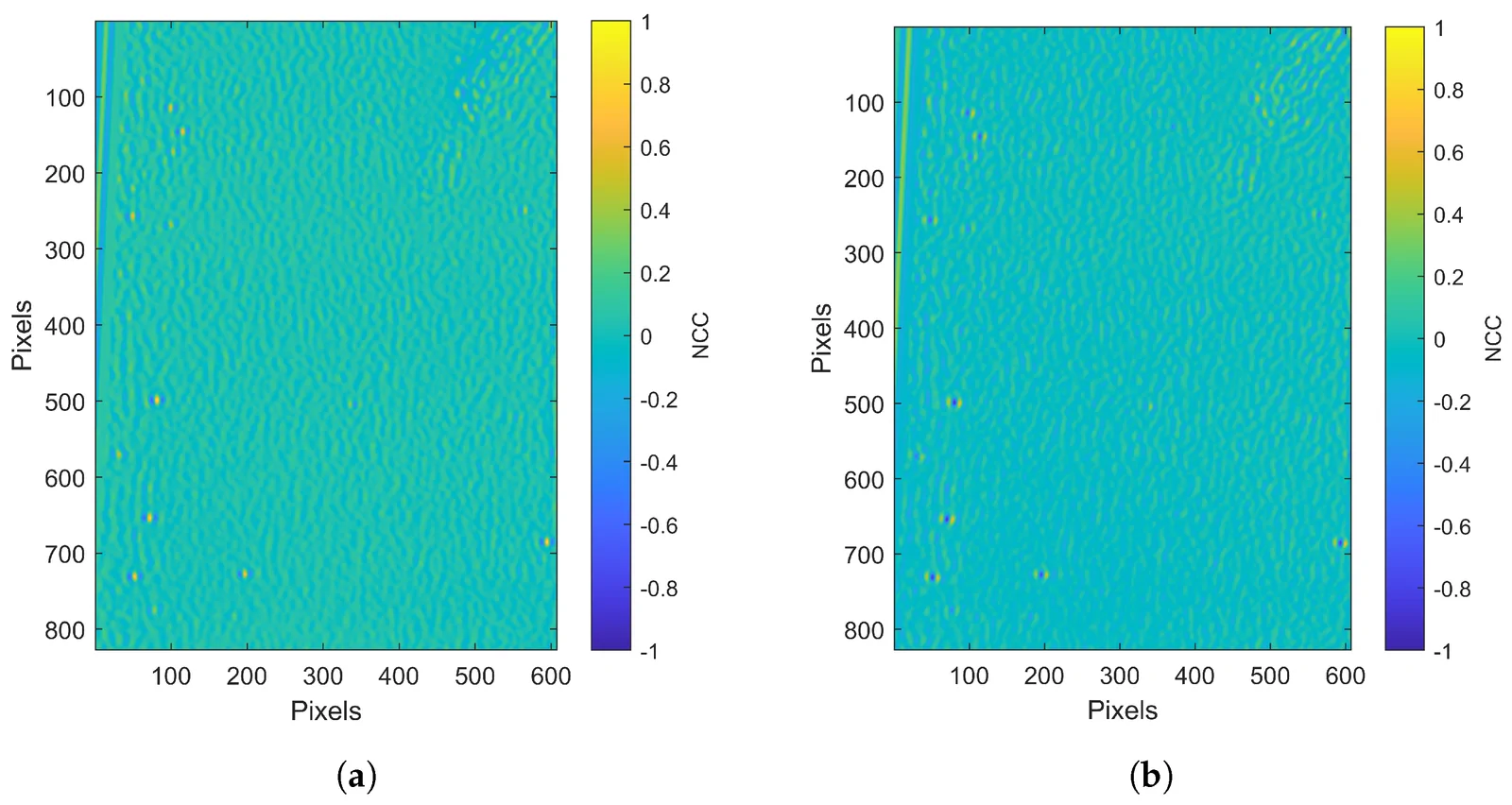
Normalized cross-correlation maps for a 606×826 pixels region of the image presented in Figure 2a with the normal template (**a**) and the flipped template (**b**).

Then, the degree of similarity between the templates and the images is evaluated using the algorithm. The NCC was implemented the MATLAB (R2024b) built-in function normxcorr2, which requires the template and the image under analysis as input arguments. The output of this normalized 2D cross-correlation function is a matrix of correlation values for each image pixel. For each image, two iterations of this algorithm were implemented, since there are two templates to evaluate. Figure 5 displays a 606×826 pixels region of the full correlation maps for the image presented in Figure 2 with the two templates presented in Figure 4.

**Figure 6:**
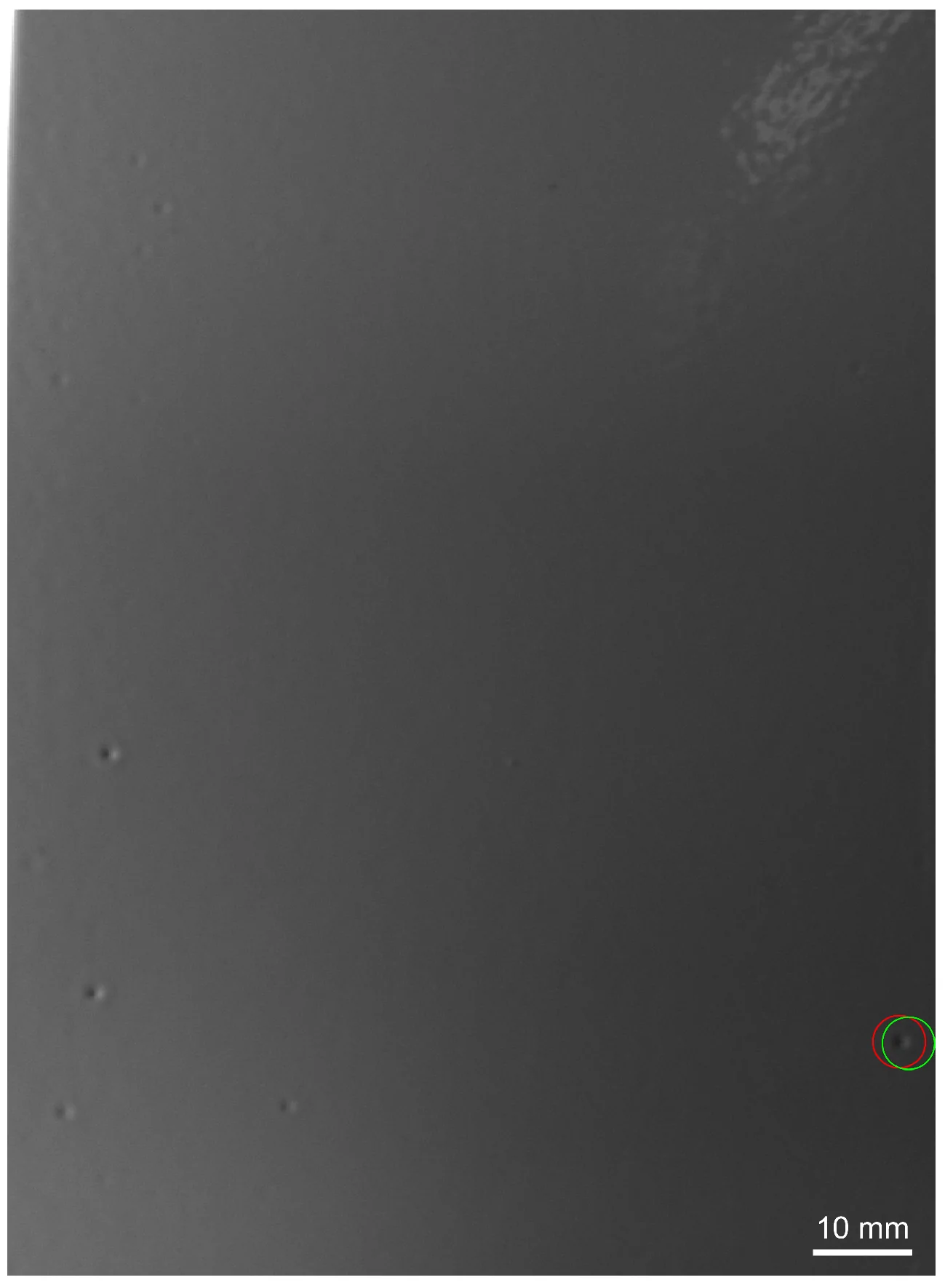
The 606×826 pixels region of the image presented in Figure 2a, with the maximum normalized cross-correlation values for the normal and flipped templates highlighted in red and green, respectively.

From the correlation map, the maximum values corresponding to the best template matches can be identified, as shown in Figure 6. The highlighted locations correspond to the maximum NCC values obtained with the templates presented in Figure 4.

With this approach, two values are generated—$X_{1}$ and $X_{2}$—for each image, corresponding to the maximum correlation values obtained with each of the two templates. Using the correlation maps presented in Figure 5 as examples, the maximum correlation value obtained was $X_{1}=0.8632$, and for the flipped template, the maximum value was $X_{2}=0.6493$. $X_{1}$ is closer to 1 than $X_{2}$, indicating that illuminating the surface from the right provides stronger evidence for the presence of a defect. However, the maximum normalized cross-correlation values alone are insufficient to reliably determine whether a defect is present. Therefore, an additional classification and decision-making step is required to improve detection performance. For this purpose, an LR model was implemented for the classification process.

### 3.3. LR Implementation

Given the binary classification objective, i.e., defective (1) vs. non-defective (0), a generalized linear model with a binomial distribution, i.e., LR, was applied. The model takes the extracted NCC-based features as its input and outputs the probability of defect presence, which is then thresholded to produce the final decision.

The proposed approach aims to develop a single model able to estimate the presence of crater defects independently of the illumination orientation in the acquired image. In this way, a composite predictor was used by combining the responses from both the original and flipped templates to ensure that the model is not dependent on the illumination orientation. Thus, the LR was implemented with a single predictor obtained by combining the two maximum values of the normalized cross-correlation ($X_{1}$ and $X_{2}$). Furthermore, a single predictor reduces model complexity and mitigates multicollinearity. Since $X_{1}$ and $X_{2}$ are derived from the same image, using normal and flipped templates, they are therefore likely to be correlated. We defined the resulting predictor $X_{3}$ according to Equation (5).

(5) $$X_{3}=X_{1}*X_{2}$$

The LR model was implemented using the MATLAB (R2024b) built-in function fitglm, with binomial distribution, using $X_{3}$ as the predictor and the binary ground-truth annotations of all images as the response variable. The model was fitted using 81 images from different water heater covers, captured using the setup described in Section 2, under different ambient lighting conditions. The dataset is well balanced, with 56% of the images showing no occurrence of defects under analysis, while 44% present at least one crater on the surface.

Table 1 presents the estimated regression coefficients and their corresponding 95% confidence intervals. Both intervals exclude zero, indicating that the intercept (${\hat{\beta }}_{0}$) and the coefficient associated with the composite predictor $X_{3}$ (${\hat{\beta }}_{1}$) are statistically significant at the 5% level.

**Figure 7:**
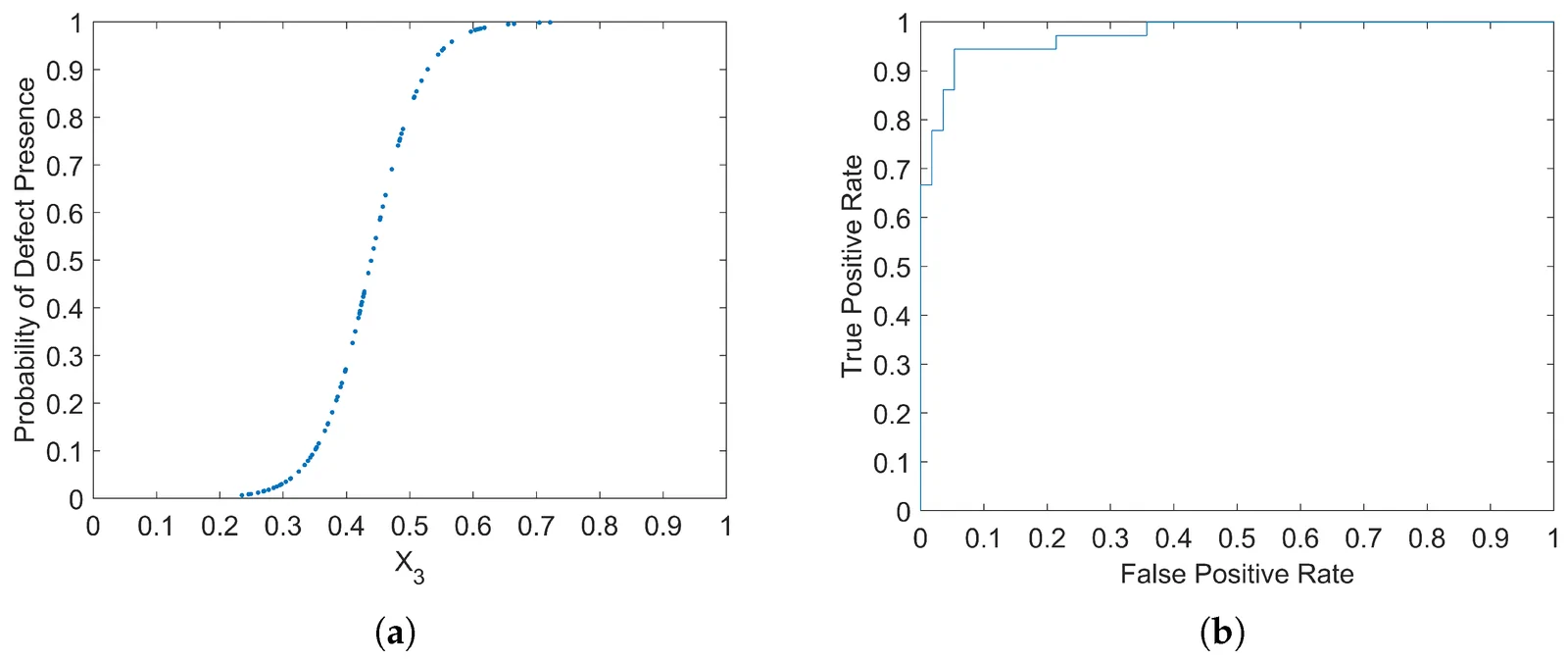
Probability of defect presence vs. the predictor $X_{3}$ (**a**). ROC curve of the proposed model with an AUC of 0.9747 (**b**).

The dependent variable, i.e., the estimated probability of the presence of the defect, was plotted against the predictor’s value ($X_{3}$), as shown in Figure 7a. As expected for a binary response, high values of $X_{3}$ tend to estimate the presence of the defect, and low values tend to estimate the non-presence of defects. Since all the obtained values of $X_{3}$ are positive, it was observed that values below 0.2 correspond to a near-zero probability of the presence of a defect and values above 0.8 indicate the presence of a crater defect. Thus, with the fitted model, we can estimate the probability of the presence of a defect in a given image.

The receiver operating characteristic (ROC) curve, presented in Figure 7b, shows that the proposed model achieves an effective discrimination between the two classes, as indicated by the area under the curve (AUC) value, 0.9747.

### 3.4. Decision Process

**Figure 8:**
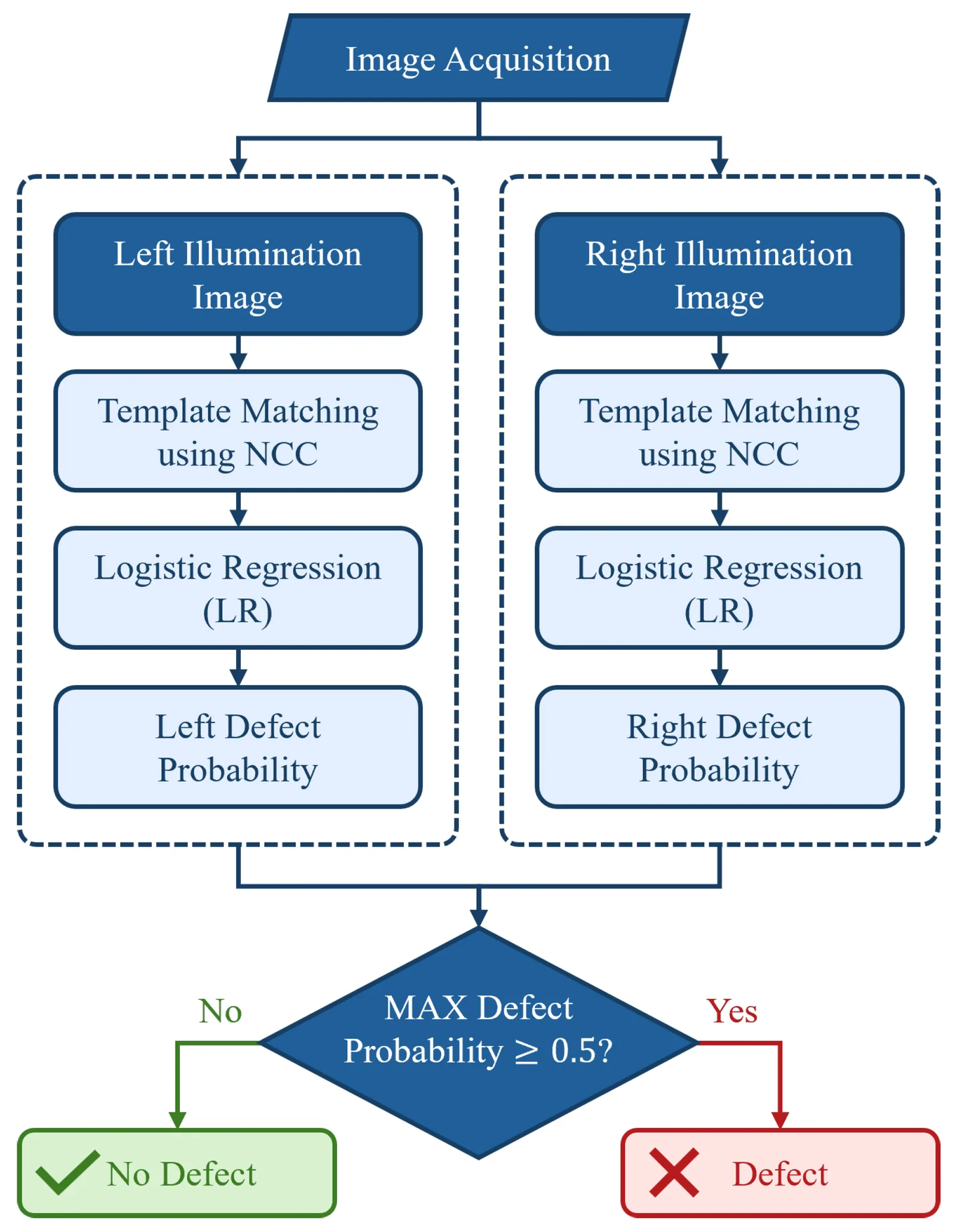
Flowchart of the proposed automatic defect-detection methodology.

As mentioned, to ensure that all of the surface is properly analyzed, two images with complementary illumination were captured for each of the water heater covers. These images are evaluated independently, producing two probabilities of defect presence, and the maximum defect probability between the two corresponding images is selected. A binary decision is then obtained by applying a probability threshold to this output, resulting in a direct indication of defect presence (or lack of defect presence) for the operator. Considering the industrial performance requirements, which prioritize minimizing false negatives, different classification thresholds were evaluated. Those within the range [0.31, 0.59] produced identical results, such as false negatives and false positives, indicating that classification performance is not affected by the selected threshold within this range. Therefore, the standard threshold of 0.5 was adopted. Figure 8 shows the flowchart of the automatic defect detection methodology proposed.

## 4. Results and Discussion

The model was evaluated using 23 water heater cover samples with different geometries. It should be noted that, due to the limited availability of defective samples in the industrial process, some physical covers were used in both the training and evaluation sets. However, the continuous movement of the covers and variations in ambient lighting result in a distinct image for each acquisition. Regarding the classification criteria, although all samples had surface defects, only those containing crater defects were considered as defective. Samples with other types of surface defects but no craters were considered as non-defective. For each sample, a pair of images was captured under different illumination orientations, right and left. The analysis of a sample, including the processing of both images and the production of a classification result, requires less than 10 s on a conventional consumer-grade laptop.

**Figure 9:**
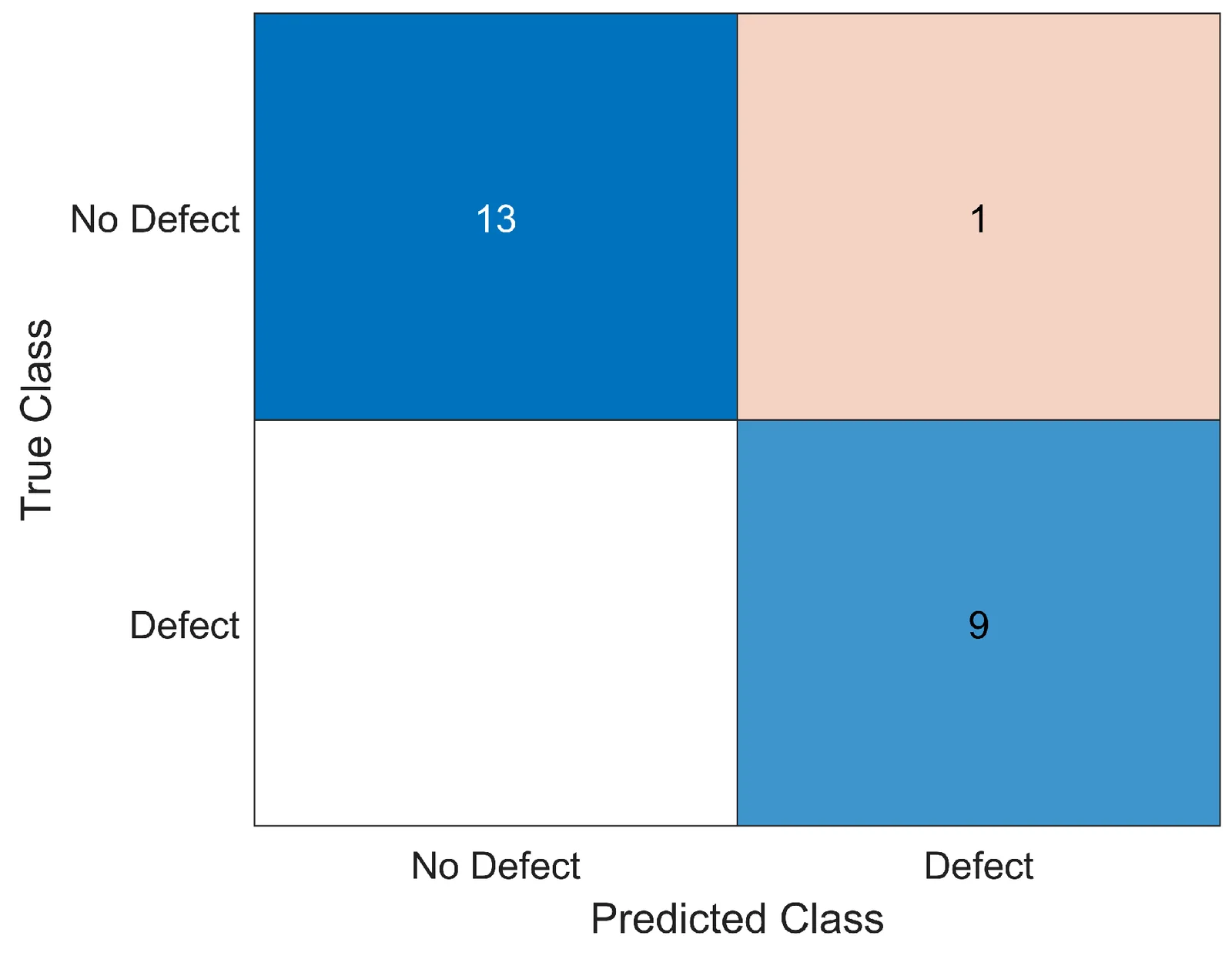
Confusion matrix for the 23 samples analyzed.

The classification results obtained for the surfaces analyzed are summarized by the corresponding confusion matrix, shown in Figure 9. These results show that the model successfully identified the presence of the crater defect in nearly all of the surfaces analyzed, with only one misclassification. The model achieved robust performance, with an accuracy of 95.65%, a precision of 90.00%, a recall of 100%, and an F1-score of 94.74%. In this application, recall is considered the most critical performance metric, since false negatives correspond to undetected defects, which can result in defective products passing the inspection stage. The achieved recall of 100% indicates that no false negatives (FN) occurred, demonstrating that the model correctly identified all real defect instances. Furthermore, only one non-defective surface was incorrectly labeled as positive (i.e., only one false positive (FP)), which is important to limit the number of unnecessary rejections.

## 5. Conclusions

This work presented the development and validation of an automated system for detecting crater defects on the painted surfaces of water heater covers, suitable for real-production-line conditions. The proposed system integrates a defect-detection model with a dark-field illumination-based image acquisition setup. The developed image acquisition system effectively captured high-quality images with clear defect visualization. The defect-detection model uses a logistic regression algorithm to estimate the probability of defect presence based on the template matching values. The final classification for each cover, indicating whether or not there is a defect, is determined based on the combined results obtained from the pair of images acquired with different illumination orientations, one per illumination side. After training, the proposed system was evaluated with 23 different water heater cover samples, achieving an accuracy of 95.65%, a precision of 90.00%, and a recall of 100%. Although the evaluation dataset was limited, the results suggest that the proposed approach is effective, particularly in terms of recall, which indicates that all defect instances were correctly identified. Overall, the proposed approach shows potential effectiveness for the inspection of the painted surfaces of water heater covers, although further validation under broader production variability in a real industrial environment is required.

## Figures and Tables

**Table 1:** Estimated coefficients and 95% confidence intervals for the LR model.

Parameter	Estimate	Std. Error	Lower CI at 95%	Upper CI at 95%
${\hat{\beta }}_{0}$	−10.82	2.46	−15.72	−5.92
${\hat{\beta }}_{1}$	24.66	5.69	13.33	35.98

## Data Availability

The data presented in this study are available on request from the corresponding author.
